# Supplementary material for: A fault-intrusion-tolerant system and deadline-aware algorithm for scheduling scientific workflow in the cloud
Source: PeerJ Comput Sci. 2021 Nov 2;7:e747. doi: 10.7717/peerj-cs.747 (PMC8576554; doi:10.7717/peerj-cs.747)
Supplement: Supplemental Information 6 [file peerj-cs-07-747-s006.zip › Myproject1.1/docs/org/cloudbus/cloudsim/VmSchedulerTimeSharedOverSubscription.html]

VmSchedulerTimeSharedOverSubscription


JavaScript is disabled on your browser.


- Overview
- Package
- Class
- Use
- Tree
- Deprecated
- Index
- Help

- Prev Class
- Next Class

- Frames
- No Frames

- All Classes

- Summary:
- Nested |
- Field |
- Constr |
- Method

- Detail:
- Field |
- Constr |
- Method


org.cloudbus.cloudsim

## Class VmSchedulerTimeSharedOverSubscription

- java.lang.Object
- - org.cloudbus.cloudsim.VmScheduler
  - - org.cloudbus.cloudsim.VmSchedulerTimeShared
    - - org.cloudbus.cloudsim.VmSchedulerTimeSharedOverSubscription

- ---

    

  ```
  public class VmSchedulerTimeSharedOverSubscription
  extends VmSchedulerTimeShared
  ```

  This is a Time-Shared VM Scheduler, which allows over-subscription. In other words, the scheduler
  still allows the allocation of VMs that require more CPU capacity that is available.
  Oversubscription results in performance degradation.

  Since:
  :   CloudSim Toolkit 3.0

- - ### Constructor Summary

    Constructors

    Constructor and Description

    `VmSchedulerTimeSharedOverSubscription(java.util.List<? extends Pe> pelist)`

    Instantiates a new vm scheduler time shared over subscription.
  - ### Method Summary

    Methods

    | Modifier and Type | Method and Description |
    | --- | --- |
    | `protected boolean` | `allocatePesForVm(java.lang.String vmUid, java.util.List<java.lang.Double> mipsShareRequested)` Allocate pes for vm. |
    | `protected void` | `redistributeMipsDueToOverSubscription()` This method recalculates distribution of MIPs among VMs considering eventual shortage of MIPS compared to the amount requested by VMs. |

    - ### Methods inherited from class org.cloudbus.cloudsim.VmSchedulerTimeShared

      `allocatePesForVm, deallocatePesForAllVms, deallocatePesForVm, getMaxAvailableMips, getMipsMapRequested, getPesInUse, setMipsMapRequested, setPesInUse, updatePeProvisioning`
    - ### Methods inherited from class org.cloudbus.cloudsim.VmScheduler

      `getAllocatedMipsForVm, getAvailableMips, getMipsMap, getPeCapacity, getPeList, getPeMap, getPesAllocatedForVM, getTotalAllocatedMipsForVm, getVmsMigratingIn, getVmsMigratingOut, setAvailableMips, setMipsMap, setPeList, setPeMap, setVmsMigratingIn, setVmsMigratingOut`
    - ### Methods inherited from class java.lang.Object

      `clone, equals, finalize, getClass, hashCode, notify, notifyAll, toString, wait, wait, wait`

- - ### Constructor Detail


    - #### VmSchedulerTimeSharedOverSubscription

      ```
      public VmSchedulerTimeSharedOverSubscription(java.util.List<? extends Pe> pelist)
      ```

      Instantiates a new vm scheduler time shared over subscription.

      Parameters:
      :   `pelist` - the pelist
  - ### Method Detail


    - #### allocatePesForVm

      ```
      protected boolean allocatePesForVm(java.lang.String vmUid,
                             java.util.List<java.lang.Double> mipsShareRequested)
      ```

      Allocate pes for vm. The policy allows over-subscription. In other words, the policy still
      allows the allocation of VMs that require more CPU capacity that is available.
      Oversubscription results in performance degradation. Each virtual PE cannot be allocated more
      CPU capacity than MIPS of a single PE.

      **Overrides:**
      :   `allocatePesForVm` in class `VmSchedulerTimeShared`

      Parameters:
      :   `vmUid` - the vm uid
      :   `mipsShareRequested` - the mips share requested

      Returns:
      :   true, if successful


    - #### redistributeMipsDueToOverSubscription

      ```
      protected void redistributeMipsDueToOverSubscription()
      ```

      This method recalculates distribution of MIPs among VMs considering eventual shortage of MIPS
      compared to the amount requested by VMs.


- Overview
- Package
- Class
- Use
- Tree
- Deprecated
- Index
- Help

- Prev Class
- Next Class

- Frames
- No Frames

- All Classes

- Summary:
- Nested |
- Field |
- Constr |
- Method

- Detail:
- Field |
- Constr |
- Method
